# Novel biconvex structure electrowetting liquid lenticular lens for 2D/3D convertible display

**DOI:** 10.1038/s41598-018-33983-x

**Published:** 2018-10-18

**Authors:** Jee Hoon Sim, Junoh Kim, Cheoljoong Kim, Dooseub Shin, Junsik Lee, Gyohyun Koo, Gyu Suk Jung, Yong Hyub Won

**Affiliations:** Convergence Optoelectronic Device Engineering (CODE) Laboratory, School of Electrical Engineering, Korea Advanced Institute of Science and Technology (KAIST), Daejeon, 34141 Republic of Korea

## Abstract

Recently, a planoconvex structure electrowetting lenticular lens capable of 2D/3D conversion through a varifocal property by an electrowetting phenomenon has been developed. However, even though it has a similar planoconvex structure to that of a commercial solid lenticular lens, comparable 3D performance could not be realized because the refractive index difference between nonconductive liquid and conductive liquid was not large. Therefore, the goal of the present study is to obtain better 3D performance compared to the conventional planoconvex structure by introducing a novel biconvex structure using ETPTA. The newly developed biconvex structure electrowetting lenticular lens showed greatly improved characteristics compared to the planoconvex structure: dioptric power (171.69D → 1,982.56D), viewing angle (26degrees → 46degrees), and crosstalk ratio (27.27% → 16.18%). Thanks to these improvements, a fine 3D image and a natural motion parallax could be observed with the biconvex structure electrowetting lenticular lens. In addition, the novel biconvex structure electrowetting lenticular lens was designed to achieve a plane lens state with a no voltage applied condition, and as such it could show a clean 2D image at 0 V. In conclusion, a novel biconvex structure electrowetting lenticular lens showed 2D/3D switchable operation as well as excellent 3D performance compared to a solid lenticular lens.

## Introduction

To develop an autostereoscopic display^1–4^ capable of 2D/3D conversion, research on the fabrication of a tunable lenticular lens using a liquid crystal^5,6^, a polymer membrane^7,8^, and a liquid lens^9,10^ has been carried out in recent years. Among these approaches, a liquid lenticular lens using an electrowetting phenomenon^11,12^ offers high optical transmittance and fast driving speed compared to the other methods. The electrowetting lenticular lens consisted of two immiscible liquids, one corresponding to conductive liquid and the other non conductive liquid. At this time, the interface between the two immiscible liquids forms a lens. If the contact angle of the conductive liquid located on the electrode is changed by applying voltage, the radius of curvature of the lens can be changed^13^. As the radius of curvature of the lens changes, the focal length also changes. Therefore, it is possible to view a 2D image when the lens is in the plane lens state and a 3D image when the lens is in the convex lens state.

According to research on the planoconvex structure electrowetting lenticular lens (P-EWLL) first reported in the literature, the dioptric power of the lenticular lens was only 145 D, and as such multi-view images are not smoothly separated from each point of view, and the ratio of crosstalk was also high^9^. In order to improve the significantly low dioptric power of the P-EWLL, research was conducted to change the material of the polymer chamber from poly methyl methacrylate (PMMA) to poly carbonate (PC), and to separate the electrode at the bottom of the chamber^10^. In addition, an opened structure chamber in which nonconductive liquid can be uniformly dosed into all lens chambers has been newly developed^14^. Through these studies, the dioptric power was improved to some extent, but it was still insufficient to reach 2,000 D, which is the dioptric power of a commercial solid lenticular lens that has fixed focal length. In this context, we propose a new electrowetting lenticular lens structure to overcome the various limitations of the P-EWLL.

**Figure 1:**
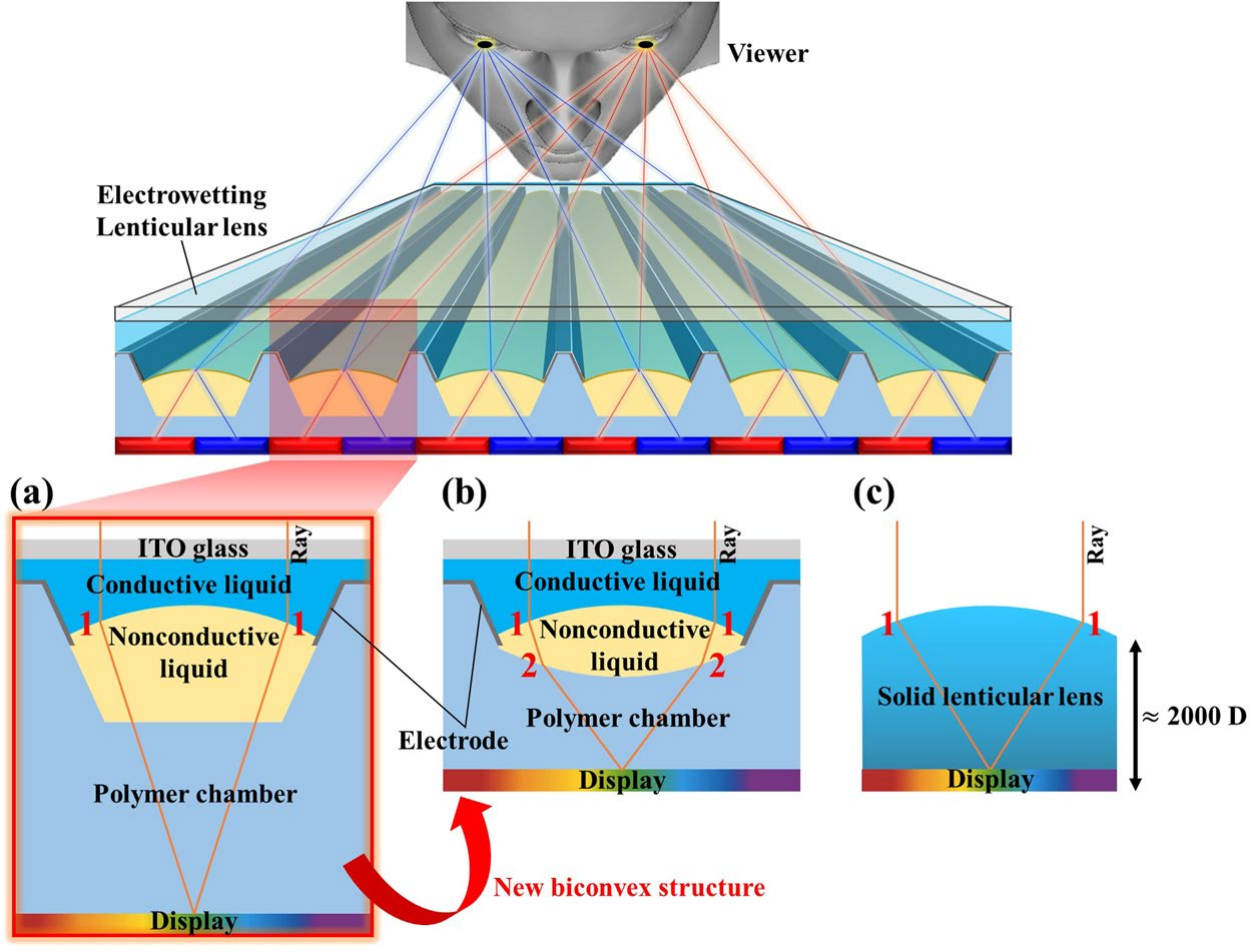
The light refraction patterns of the (**a**) planoconvex structure, (**b**) biconvex structure, and (**c**) commercial solid lenticular lenses (The numbers 1 and 2 indicate the sequence of refraction of light.).

As presented in Fig. 1a, it was confirmed that the refraction of light occurs only once at the interface between the conductive liquid and the nonconductive liquid in the P-EWLL. The refraction of light occurs only once in the case of the solid lenticular lens as well, as can be seen in Fig. 1c. However, the commercial solid lenticular lens has a shorter focal length than that of the P-EWLL. This is because the refractive index difference between the nonconductive liquid and the conductive liquid in the electrowetting lenticular lens system is relatively smaller than the refractive index difference between the solid lenticular lens and the air. For this reason, even when the two lenticular lenses have similar structures, the focal length of the solid lenticular lens is much shorter than that of the P-EWLL. However, as mentioned above, when the lenticular lens has a low dioptric power, the multi-view images are not smoothly separated, and the ratio of crosstalk also increases. Therefore, it is necessary to fabricate an electrowetting lenticular lens with the same dioptric power as the solid lenticular lens in Fig. 1c while overcoming the refractive index difference between nonconductive liquid and conductive liquid. In order to meet the aforementioned requirements, a novel biconvex structure electrowetting lenticular lens (B-EWLL) capable of further refracting light, which is shown in Fig. 1b, is proposed in this study.

## Conditions of a biconvex structure electrowetting lenticular lens for the plane lens state at no applied voltage

**Figure 2:**
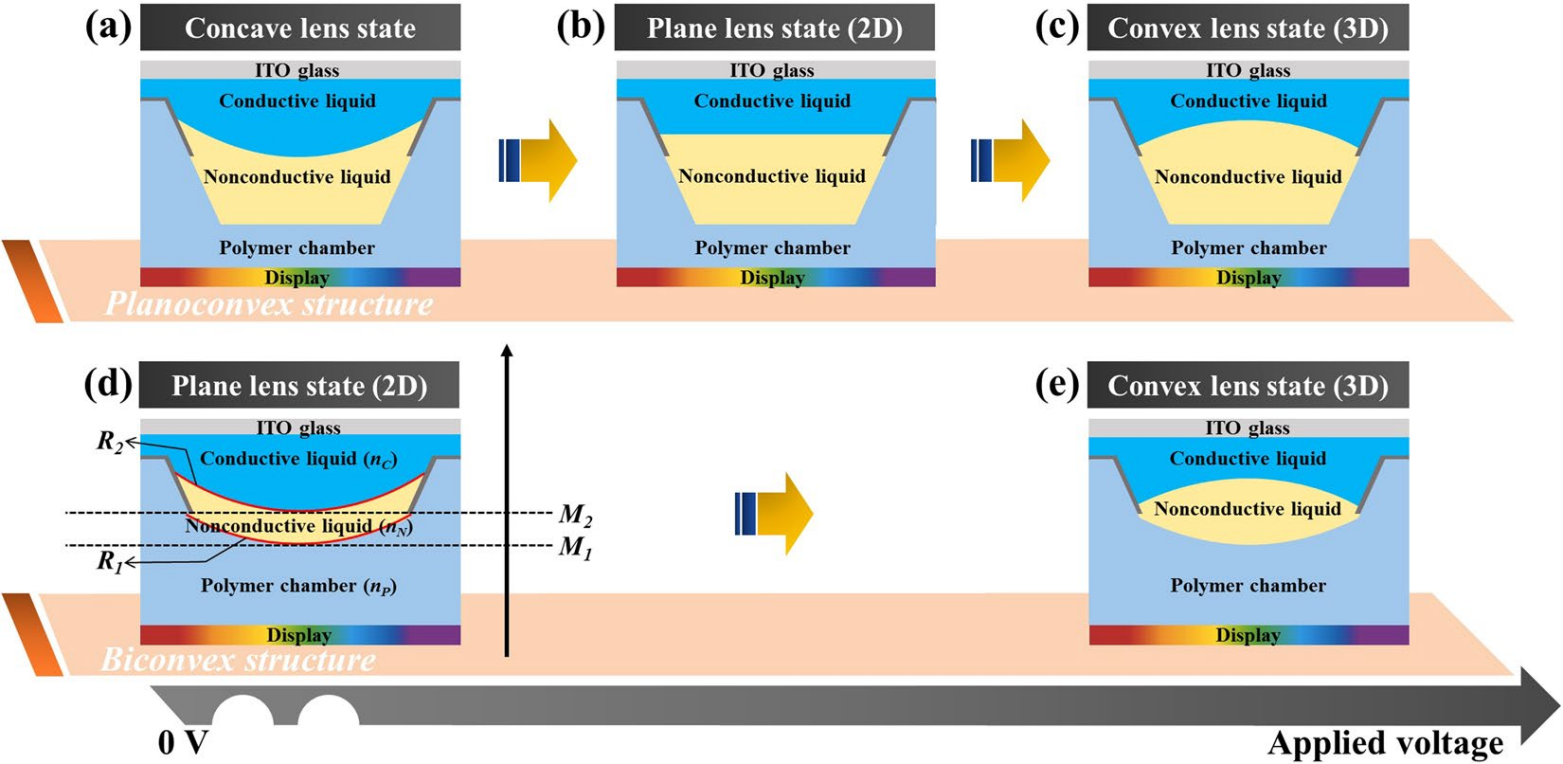
Lens shapes of the (**a**–**c**) planoconvex structure and (**d**–**e**) biconvex structure electrowetting lenticular lenses according to applied voltage.

The lens shapes of the B-EWLL are shown in Fig. 2d,e. For an effective comparison, the cases of the conventional P-EWLL are also presented in Fig. 2a–c. Using the electrowetting phenomenon, the P-EWLL can be changed from a concave lens state to a convex lens state as shown in Fig. 2a–c. In addition, the B-EWLL also controls the interface between the two immiscible liquids in the same manner as shown in Fig. 2d,e. However, since the bottom surface of the chamber is not a plane but a spherical surface, the lens characteristics are different from those of the P-EWLL. For example, Fig. 2d is not necessarily a concave lens state. It is apparent that the lens state in Fig. 2e has higher dioptric power than that of Fig. 2d. In turn, it is necessary to establish a condition in Fig. 2d corresponding to the initial state in which no voltage is applied. Since the radius of curvature of the boundary between the conductive liquid and the nonconductive liquid is determined, the lens state can become a concave lens state or a plane lens state. Therefore, an ABCD matrix analysis was applied to the corresponding radius of curvature so that the lens state in Fig. 2d would form a plane lens state.

Figure 2d shows a cross-sectional view of the B-EWLL with no voltage applied. In this case, *R*_1_ is the radius of curvature of the bottom boundary of the non conductive liquid (this value is determined by the structure of the chamber), and *R*_2_ is the radius of curvature of the upper boundary of the nonconductive liquid where no voltage is applied. The refractive index of each material is expressed as *n*_*P*_, *n*_*N*_, and *n*_*C*_, which in turn corresponds with the refractive index of the polymer chamber, nonconductive liquid, and conductive liquid, respectively. Based on these relations, an ABCD matrix analysis for the this optical system was performed as delineated in equations (1–2). *M*_1_ and *M*_2_ denote the ABCD matrix at the bottom and upper boundaries of the nonconductive liquid, respectively.

1 $$M={M}_{2}\times {M}_{1}$$

2 $$\therefore M=[\begin{array}{cc}1 & 0\\ \frac{{n}_{N}-{n}_{C}}{{R}_{2}\times {n}_{C}} & \frac{{n}_{N}}{{n}_{C}}\end{array}]\times [\begin{array}{cc}1 & 0\\ \frac{{n}_{P}-{n}_{N}}{{R}_{1}\times {n}_{N}} & \frac{{n}_{E}}{{n}_{N}}\end{array}]=[\begin{array}{cc}1 & 0\\ \frac{{n}_{N}-{n}_{C}}{{R}_{2}\times {n}_{C}}+\frac{{n}_{P}-{n}_{N}}{{R}_{1}\times {n}_{C}} & \frac{{n}_{P}}{{n}_{C}}\end{array}]\cong [\begin{array}{cc}1 & 0\\ -\frac{1}{f} & 1\end{array}]$$

In order for the lenticular lens to have the same effect as the plane lens, the focal length *f* must be infinite, and hence the second row and first column of the ABCD matrix *M* must be zero. Therefore, the following can be expressed as equations (3–4).

3 $$\frac{{n}_{N}-{n}_{C}}{{R}_{2}\times {n}_{C}}+\frac{{n}_{P}-{n}_{N}}{{R}_{1}\times {n}_{C}}=0$$

4 $$\frac{{R}_{2}}{{R}_{1}}=\frac{{n}_{N}-{n}_{C}}{{n}_{N}-{n}_{P}}$$

In conclusion, the B-EWLL can exhibit the same effect as the plane lens in the initial state when the radius of curvatures *R*_1_ and *R*_2_ satisfy equation (4).

## Fabrication process of a biconvex structure electrowetting lenticular lens

**Figure 3:**
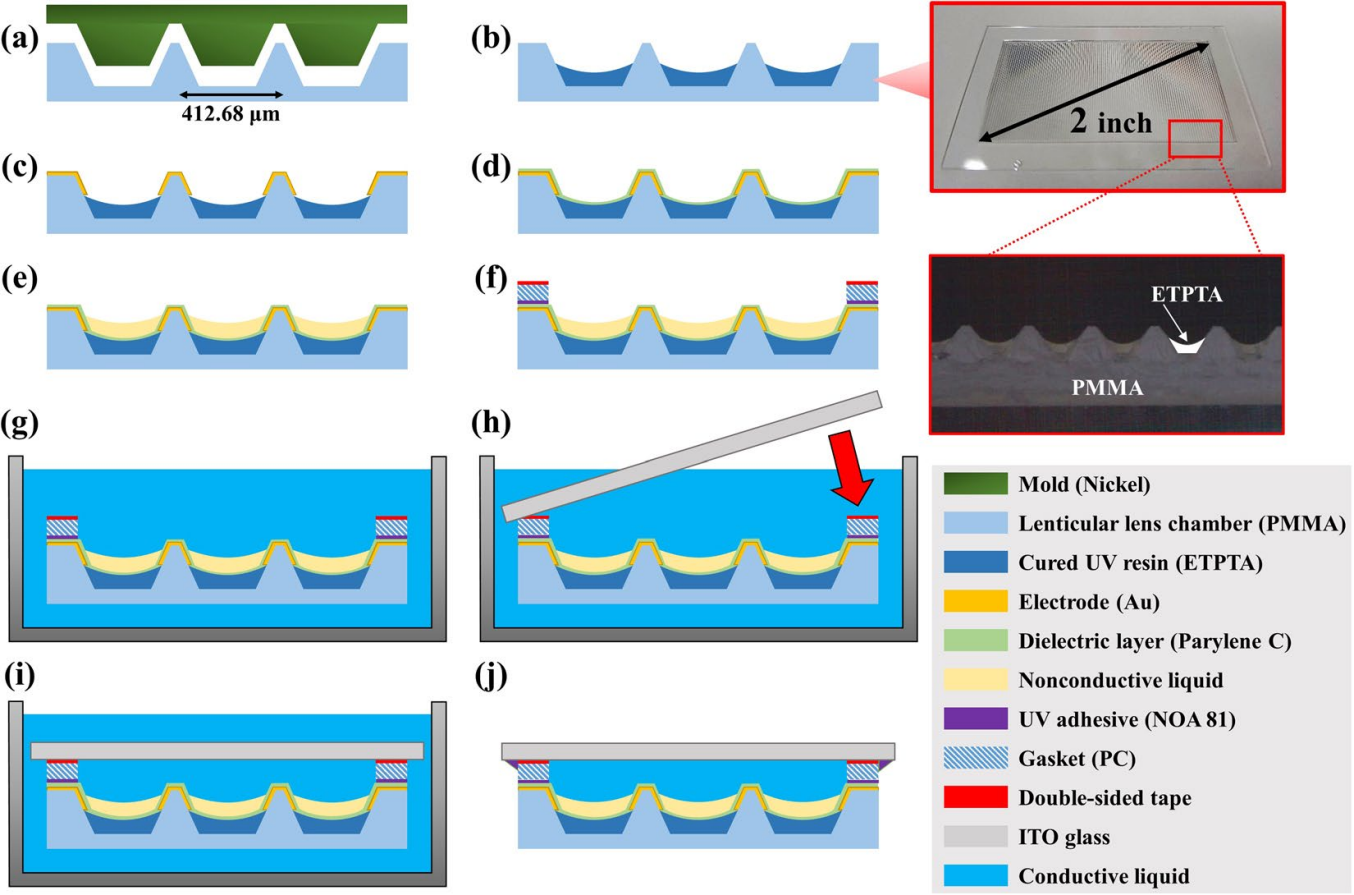
Fabrication process of a biconvex structure electrowetting lenticular lens. (**a**) A PMMA lenticular lens chamber manufactured by hot embossing process. (**b**) ETPTA UV resin dosing and curing (in the case of P-EWLL, this procedure is omitted). (**c**) Au electrode deposition. (**d**) Parylene C dielectric layer deposition. (**e**) Nonconductive liquid dosing. (**f**) Fixation of gasket through UV adhesive. (**g**) Immersing the lenticular lens chamber in the conductive liquid bath. (**h**–**i**) In the bath, ITO glass was bonded to the gasket using double-sided tape. (**j**) A completed biconvex structure electrowetting lenticular lens.

First, the polymer lenticular lens chamber is fabricated through pitch selection, KOH wet etching of silicon, nickel electro-plating, and a hot embossing process^9^. The hot embossing process is shown in Fig. 3a. At this time, the polymer lenticular lens chamber has a 412.68 μm pitch, determined according to the G pro 2 model, a smartphone launched by LG Electronics, and has a lens area of 2 inches in total^9^. Since the number of pixels of the G pro 2 display corresponding to this lens pitch is six, it is designed to be able to realize up to six viewpoints. Next, in order to form a biconvex structure, the bottom of the chamber should have a curved surface. Therefore, the mass production method of UV-curing after uniformly dosing the ethoxylated trimethylolpropane triacrylate (ETPTA) was applied to the PMMA chamber manufactured through the hot embossing process, as shown in Fig. 3b. ETPTA is a UV resin made by mixing monomer, trimethylolpropane ethoxylate triacrylate, and an initiator, 2-Hydroxy-2-methylpropiophenone, in a ratio of 6: 1. At this time, since the refractive index of ETPTA (*n*_*E*_ = 1.47) and the refractive index of PMMA (*n*_*P*_ = 1.48) do not show a large difference, it is assumed that light refraction rarely occurs at the boundary between the two materials. After dosing and curing of ETPTA, 60 nm Au (gold) electrodes were deposited by thermal evaporation on both sides^10^, as shown in Fig. 3c. Once the deposition of the electrodes is complete, the presence of a dielectric layer is essential to enable the electrowetting lenticular lens to function as a stable electrowetting-on-dielectric (EWOD) device. Therefore, the chemical vapor deposition (CVD) method was applied at room temperature, and Parylene C was deposited for a dielectric layer, as shown in Fig. 3d. In addition, Parylene C has an oleophilic property, and therefore the process of dosing nonconductive liquid in a chamber can be carried out under atmospheric conditions. The thickness of the Parylene C dielectric layer was 1 μm. After completing dosing of the nonconductive liquid, as shown in Fig. 3e, the lenticular lens chamber had to be sealed with DI water. At this time, the volume of the nonconductive liquid whose radius of curvature of the upper boundary *R*_2_ satisfies equation (4) was calculated and dosed into the chamber. The sealing process was carried out in a water bath, as shown in Fig. 3g–i, and the inside of the sample was completely filled with DI water. Finally, the UV adhesive (NOA 81) was cured on the ITO glass and gasket boundary, and the sealing process was thus completed, as shown in Fig. 3j.

**Figure 4:**
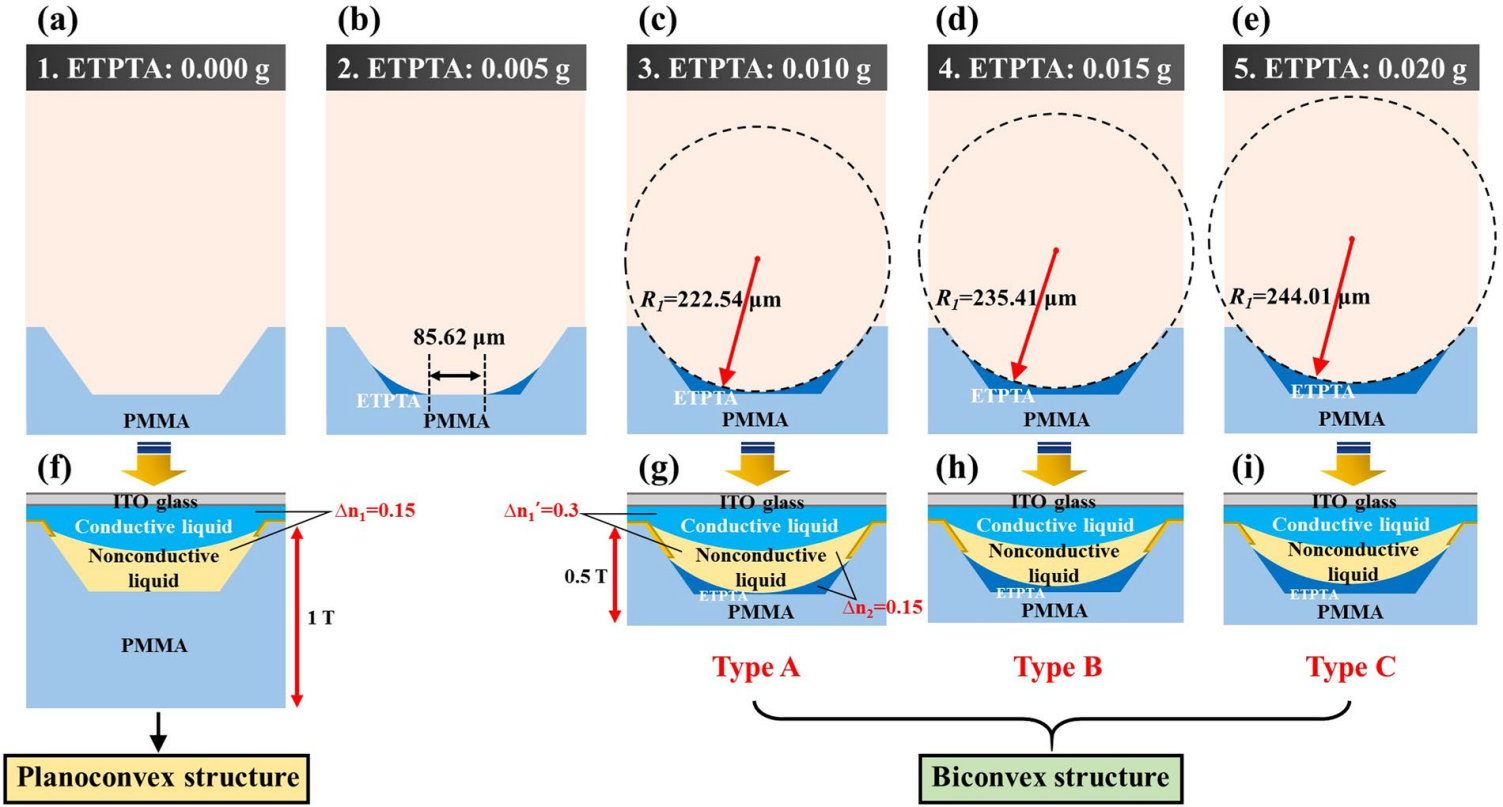
Cross-sectional diagrams of PMMA lenticular lens chambers dosed ETPTA (**a**) 0.000 g, (**b**) 0.005 g, (**c**) 0.010 g, (**d**) 0.015 g, (**e**) 0.020 g, and Cross-sectional diagrams of electrowetting lenticular lenses: (**f**) P-EWLL, (**g**–**i**) B-EWLL (Type A to C in order).

In this research, we tried to form a spherical boundary on the bottom of the chamber by dosing ETPTA. When ETPTA was injected into a 2 inch PMMA lenticular lens chamber through a Musashi dispenser, the minimum dosed weight was 0.005 g. Therefore, the amount of ETPTA dosed in the PMMA lenticular lens chamber was classified into five cases (0.000 g, 0.005 g, 0.010 g, 0.015 g, and 0.020 g). Figure 4a–e are cross-sectional views showing changes in the PMMA lenticular lens chamber according to the dosed weight of ETPTA (in each case, the results of measuring the transverse-direction step of the chamber by α-step are provided in Figure S1 on the Supplementary Information). A reverse-trapezoidal shape empty chamber, as shown in Fig. 4a, was used for P-EWLL fabrication. Thereafter, except for the case where 0.005 g of ETPTA was dosed, ETPTA became a complete spherical boundary after UV curing as shown in Fig. 4c–e. When 0.005 g of ETPTA was dosed, the bottom of the chamber did not form a spherical boundary (see Fig. 4b) because there was about an 85.62 μm region where ETPTA was almost absent. In addition, as shown in Fig. 4c–e, the radius of curvature *R*_1_ of the cured ETPTA spherical boundary increases slightly as the amount of ETPTA dosed onto the PMMA lenticular lens chamber increases. Theoretically, since the surface tension between ETPTA, PMMA, and vapor is constant, the contact angle of ETPTA is also constant, and thus *R*_1_ should be constant. However, in reality, as the dosed amount of ETPTA increases, the influence of gravity also increases to some extent, and hence the contact angle of ETPTA increases slightly, resulting in an increase in *R*_1_. Therefore, in this research, only the cases of dosing ETPTA two times (0.010 g), three times (0.015 g), and four times (0.020 g) in the PMMA lenticular lens chamber were considered.

In the case of the P-EWLL, a 1 T thick PMMA lenticular lens chamber was used, as in previous research^9^. In addition, the nonconductive liquid with a refractive index matching the PMMA lenticular lens chamber was used to prevent a prism effect at some of the partition wall where the electrodes were not covered. On the other hand, in the case of the B-EWLL, since light refraction was also considered between the nonconductive liquid and the chamber, the refractive indices of the two materials should be different. As a result, a nonconductive liquid with a higher refractive index than that of PMMA was used for the B-EWLL. Finally, it is considered that the B-EWLL will be able to realize higher dioptric power than the P-EWLL by the same voltage, and therefore a 0.5 T PMMA lenticular lens chamber is used. Hereinafter, the conditions such as the type of the conductive liquid, the kind of the electrode material, and the thickness of the gasket are all applied equally. The characteristics of the various types of electrowetting lenticular lenses are listed in Table 1 for comparison. In addition, cross-sectional views of each type of electrowetting lenticular lens are shown in Fig. 4f–i.

**Table 1:** Specifications of electrowetting lenticular lenses (No. 1: P-EWLL, No. 2: Not a lens, and No. 3~5: B-EWLL).

No.	Quantity of ETPTA [g]	ROC of ETPTA [μm]	Volume of NC [μl]	Structure	Remarks
1	0.000		37.00	Planoconvex	—
2	0.005				—
3	0.010	222.54	59.05	Biconvex	Type A
4	0.015	235.41	45.71	Biconvex	Type B
5	0.020	244.01	39.45	Biconvex	Type C

(NC: Nonconductive liquid).

Through Fig. 4f–i, the visible differences of the lenses can be more easily identified. Figure 4f shows that the sample of the P-EWLL has a thickness of 1 T in the chamber, and the refractive index difference Δ*n*_1_ between the nonconductive liquid and the conductive liquid is 0.15. On the other hand, for the biconvex structure of Type A to C, the thickness of the chamber is 0.5 T, and the refractive index difference Δ*n*_1_′ between the nonconductive liquid and the conductive liquid is 0.3 (a nonconductive liquid with a higher refractive index than that of P-EWLL was used), as shown in Fig. 4g–i. In addition, in the case of the B-EWLL, there is a difference Δ*n*_2_ in the refractive index between the chamber material and the nonconductive liquid, and therefore the difference Δ*n*_2_ is 0.15.

## Results of dioptric power measurement

**Figure 5:**
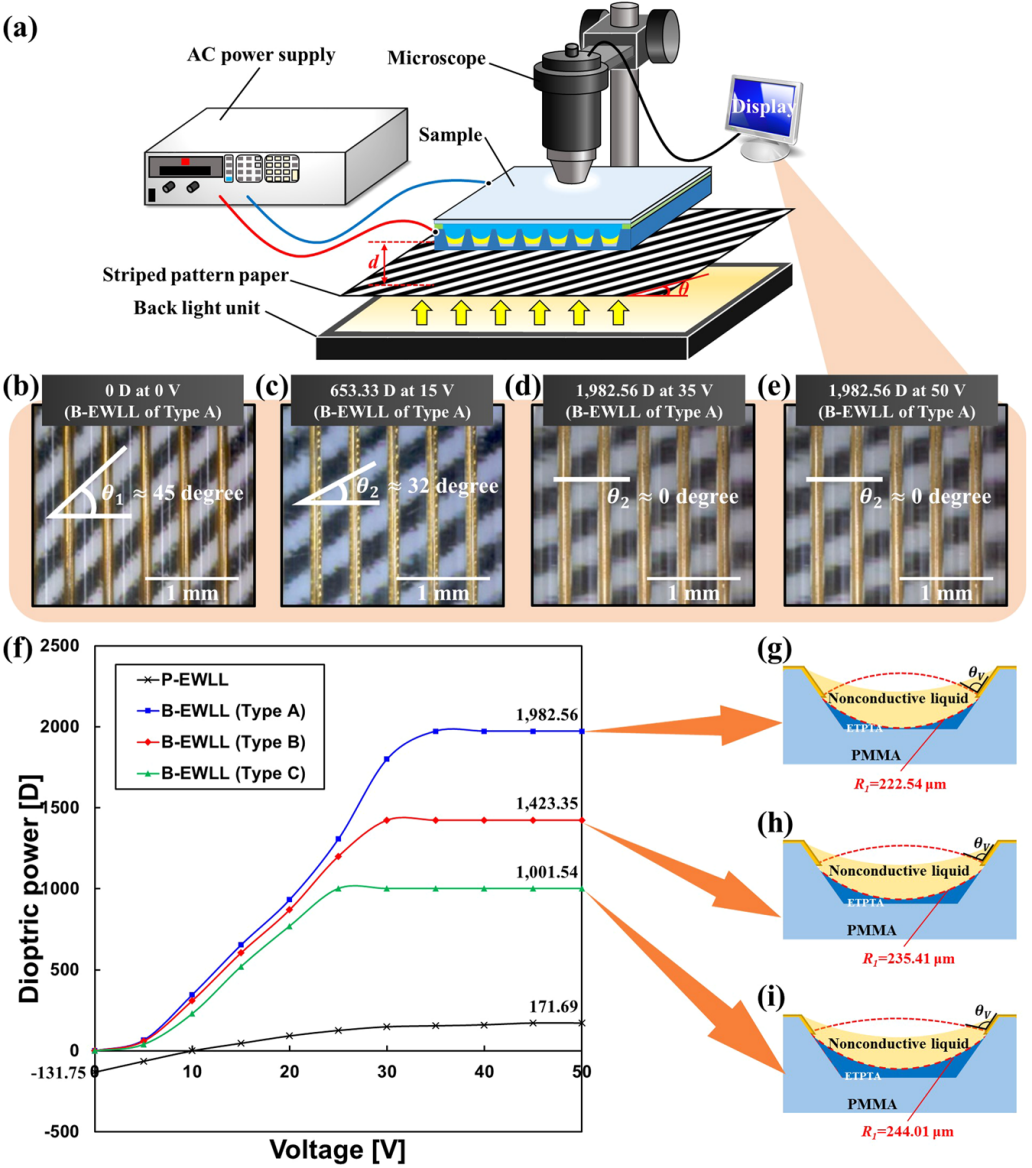
(**a**) A schematic diagram of the dioptric power measurement method of an electrowetting lenticular lens, and microscopic images of the black striped pattern when the dioptric power of the Type A sample of the B-EWLL is (**b**) 0 D, (**c**) 653.33 D, and (**d**–**e**) 1,982.56 D. (**f**) is a graph showing the dioptric power measurement results of P-EWLL and B-EWLL samples. (**g**,**h**,**i**) correspond to cross-sectional diagrams of Type A, B, and C samples of the B-EWLL, respectively (the red dotted line indicates when the lens has reached its maximum dioptric power).

Dioptric power is the most important factor for measuring the optical power of a lens application, and can be used to quantitatively interpret the quality of a three-dimensional image^15^. To measure the dioptric power of the fabricated electrowetting lenticular lens, the completed sample was placed on black stripe patterned paper, as shown in Fig. 5a. A back light unit (BLU) emitting white light was placed under this paper. Therefore, the measurement method was set so that the light emitted from the BLU sequentially passes through the paper and the sample and reaches the microscope. Finally, an electrode was connected to the chamber of the electrowetting lenticular lens and the ITO glass from the AC power supply, respectively. The width and height of the striped pattern continuously change from the plane lens state to the convex lens state depending on the voltage in the sample. Therefore, it can be seen that the angle becomes smaller as the lens becomes convex (see Fig. 5b–e).

The dioptric power of the electrowetting lenticular lens can be expressed by equation (5) below.

5 $$D=\frac{1}{f}=(1-\frac{1}{M})\frac{1}{d}$$

where *f*, *M*, and *d* represent the focal length, magnification, and distance between the striped pattern paper and electrowetting lenticular lens, respectively. Through Fig. 4f–i, *d* can be 0.5 T or 1 T depending on the case, and the magnification *M* is usually calculated from the image size changes as given in equation (6).

6 $$M=\frac{\tan \,{\theta }_{1}}{\tan \,{\theta }_{2}}$$

where *θ*_1_ is the angle of the black striped pattern when the lens is plane state, and *θ*_2_ is the angle of black striped pattern when various voltages are applied. In the lenticular lens system, the magnification *M* occurs only on the horizontal scale, and the vertical scale is constant even when voltage is applied. This is because the curvature of each electrowetting lenticular lens changes with respect to the horizontal direction, but is constant with respect to the vertical direction. Therefore, if the shape of the lens becomes convex, the refraction of the black striped pattern under the lenticular lens array occurs, and the pattern is magnified in the horizontal direction. When the vertical scale is fixed, the magnification *M* can be calculated from equation (6) and the dioptric power *D* can be calculated from equation (5) since the rate of change of length is the same as the rate of change of the tangent angle.

Based on the measurement method described above, the graph of the dioptric power of the electrowetting lenticular lenses corresponding to P-EWLL and B-EWLL in Fig. 4f–i is summarized in Fig. 5f. The dioptric power was measured in a voltage range from 0 V to 50 V.

First, in the case of the P-EWLL, dioptric power of −131.75 D was shown at 0 V. Since it corresponds to the concave lens state until the plane lens is formed at 10 V, a 2D image cannot be seen in this section. Then, as the voltage increases, the dioptric power gradually increases. At a voltage of 45 V, saturation occurs and the dioptric power corresponds to 171.69 D. Next, in the case of the B-EWLL of Type A to C, 0 D was shown in a state of 0 V. As a result, all of the Type A to C samples correspond to a state where a clean 2D image can be initially viewed. In addition, for the B-EWLL of Type A to C, it was confirmed that the dioptric power according to voltage was generally higher than that of the P-EWLL. The reason for this is that the refractive index difference between the nonconductive liquid and the conductive liquid is different between the P-EWLL and the B-EWLL (${\rm{\Delta }}{n^{\prime} }_{1}-{\rm{\Delta }}{n}_{1}=0.15$). Furthermore, in the case of the B-EWLL, the dioptric power was increased because refraction occurred at the boundary between the chamber material and the nonconductive liquid (${\rm{\Delta }}{n}_{2}=0.15$).

Next, Fig. 5g–i show the reason why there is a maximum dioptric power difference between Type A to C. From Fig. 5g–i, it was confirmed that the amount of ETPTA dosed in the PMMA lenticular lens chamber increases. At this time, the radius of curvature *R*_1_ increases slightly as the amount of ETPTA in the chamber increases. In addition, since the exposed portions of the partition walls not covered with the ETPTA are reduced, the width at which the electrode can be deposited is gradually decreased. Since the electrode width is reduced, the moving range of the conductive liquid by the electrowetting phenomenon is also reduced, and thus the contact angle *θ*_*V*_ of the conductive liquid by the voltage reaches the saturation state more quickly. This means that as the amount of ETPTA increases, the final contact angle *θ*_*V*_ of the conductive liquid increases. In other words, the increase in the contact angle *θ*_*V*_ of the conductive liquid corresponds with an increase in the radius of curvature at the interface between the conductive liquid and the nonconductive liquid. In conclusion, as the amount of ETPTA in the chamber increases, the radius of curvature at both boundaries of the nonconductive liquid increases. As a result, the Type A sample with the least amount of ETPTA in the chamber was advantageous to realize a high dioptric power because the radius of curvature of both boundaries of the nonconductive liquid was smaller than the other two samples. This can be easily seen by comparing the shape of the lens portion indicated by the red dotted line in Fig. 5g–i. The maximum dioptric power of Type A to C is 1,982.56 D, 1,423.35 D, and 1,001.54 D, respectively, as shown in Fig. 5f. Thus, the Type A sample among the B-EWLLs fabricated in this research showed the highest dioptric power. Therefore, in this research, the Type A sample was utilized and analyzed as a representative B-EWLL. First, the maximum dioptric power of the Type A sample was 1,982.56 D, which was 1,810.87 D higher than the maximum dioptric power of the P-EWLL. As described above, the Type A sample of the B-EWLL showed a dioptric power of 0 D when no voltage was applied. This means that 2D images can be viewed without additional voltage and voltage of 10 V is saved compared to the P-EWLL in a 2D state. In conclusion, the electrowetting lenticular lens changed from a planoconvex structure to a biconvex structure, which initially satisfied the plane lens state (2D state) and lowered the operating voltage. In addition, the dioptric power was greatly improved.

## Results of viewing angle and crosstalk measurement

To measure the viewing angle and crosstalk of the electrowetting lenticular lens, a simple two-view image test was performed (a schematic diagram of the viewing angle and crosstalk measurement is provided in Figure S2 of the Supplementary Information). First, the electrowetting lenticular lens sample was placed on the LCD. A two-view image that alternates red and green patterns was then displayed on the LCD. Since six pixels in the pitch of the lenticular lens cell are designed to correspond to each other, two-view images were produced by allocating three pixels to each of the red and green patterns. A microscope was then placed at a distance *D* from the sample, where *D* was 300 mm as the observation distance. The microscope is connected to the rotator and is designed to rotate by the given rotation angle *θ* (from −25 degrees to +25 degrees). Electrowetting lenticular lens samples were supplied through an AC power supply. Intensity of red light and green light at each rotation angle was measured when the sample assumed a convex lens state.

**Figure 6:**
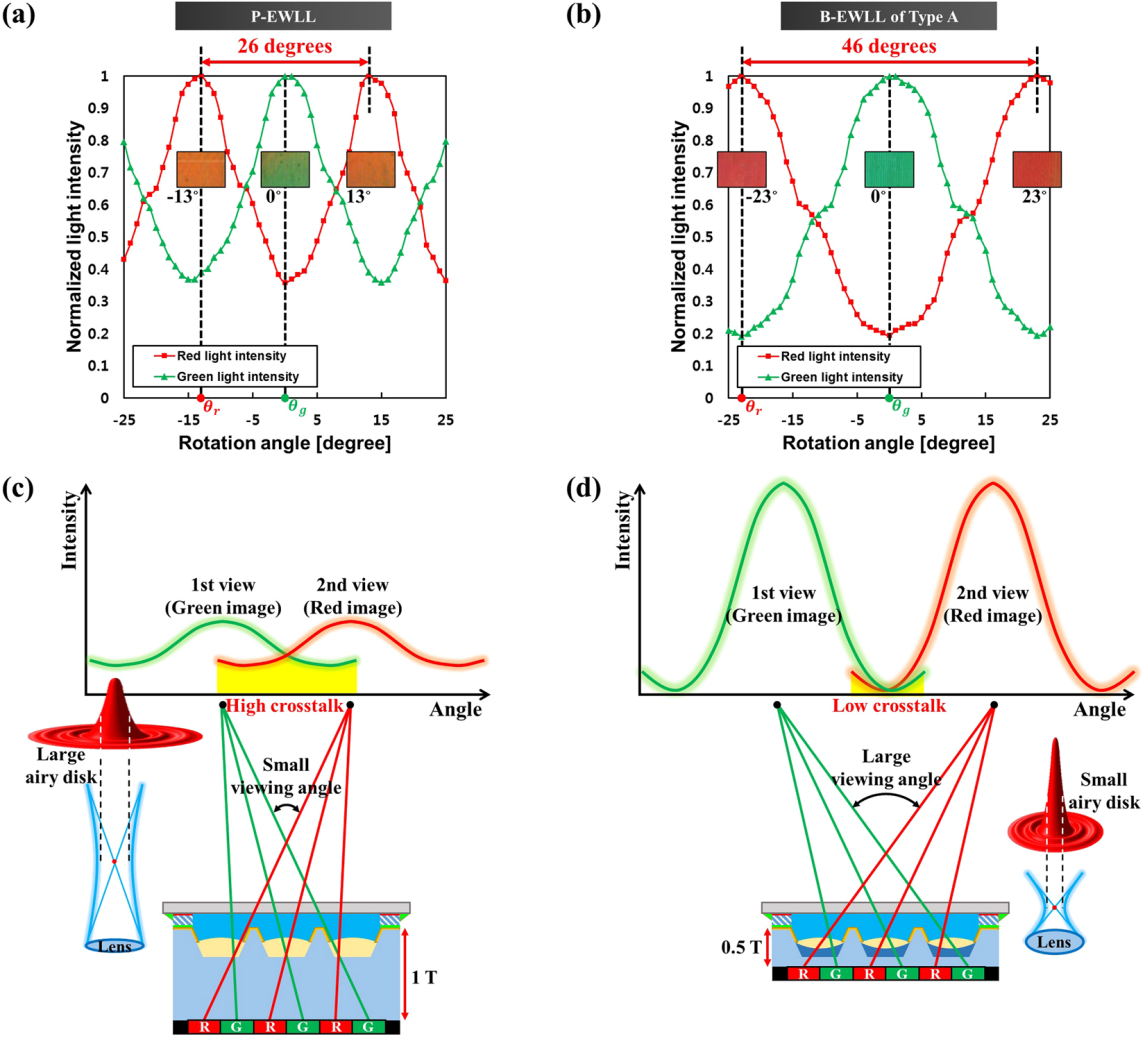
Graphs showing normalized light intensity according to rotation angle of (**a**) the P-EWLL and (**b**) B-EWLL of Type A. Microscopic images at the points where the intensity of red and green is highest are shown in each graph. Schematic diagrams of crosstalk of red and green light in (**c**) the P-EWLL and (**d**) B-EWLL of Type A.

Figure 6a,b show the results of light intensity measurement according to the rotation angle of the P-EWLL and the B-EWLL of Type A. At this time, the measurement was carried out with a dioptric power of 171.69 D for the P-EWLL and 1,982.56 D for the Type A B-EWLL. In this research, the viewing angle is defined as the angle difference between different red light intensity peaks. Therefore, the viewing angles in each case were found to be 26 degrees (−13 degrees and +13 degrees) in the P-EWLL and 46 degrees (−23 degrees and +23 degrees) in the B-EWLL. As a result, the B-EWLL had a wider viewing angle than that of the P-EWLL. For this reason, since the thickness of the PMMA lenticular lens chamber of the B-EWLL was reduced from 1 T to 0.5 T, the distance between the image and the optical center of the lenticular lens can be greatly reduced. This effect increased the angle of separation between the red and green images due to the extension of the viewing angle.

Next, the crosstalk of the P-EWLL and the B-EWLL was calculated through equation (7).

7 $${\rm{Crosstalk}}=\frac{1}{2}\times \{\frac{{I}_{r}({\theta }_{g})}{{I}_{r}({\theta }_{g})+{I}_{g}({\theta }_{g})}+\frac{{I}_{g}({\theta }_{r})}{{I}_{r}({\theta }_{r})+{I}_{g}({\theta }_{r})}\}$$

In this case, *θ*_*r*_ and *θ*_*g*_ indicate the rotation angles when the red light intensity and the green light intensity show the maximum values in the main field of view (Fig. 6a,b show *θ*_*r*_ and *θ*_*g*_). *I*_*r*_(*θ*) and *I*_*g*_(*θ*) denote the light intensity of red and green light at the rotation angle *θ*, respectively. The crosstalk ratio of the P-EWLL and the Type A B-EWLL calculated using equation (7) were 27.27% and 16.18%, respectively. Thus, the B-EWLL had a smaller ratio of crosstalk than that of the P-EWLL. The reasons for these results are demonstrated with Fig. 6c,d.

First, the maximum dioptric power of the B-EWLL was 1,982.56 D, which was 1,810.87 D higher than the maximum dioptric power of the P-EWLL. The higher dioptric power means that the distance between the image and the optical center of the lenticular lens is shorter, which prevents diffraction of the light and makes the airy disk of light passing through the lens smaller. To explain this in more detail, the following numerical aperture (NA) equation (8) should be noted.

8 $${\rm{Numerical}}\,{\rm{aperture}}\,({\rm{NA}})=\frac{nD}{2f}$$

where *n* is the refractive index of the lens, *D* is the diameter of lens, and *f* is the focal length of the lens. That is, as the dioptric power of the lens increases, the focal length *f* becomes shorter, which leads to an increase in NA. The relationship between NA and the airy disk is delineated by equation (9) below.

9 $$r=0.61\times \frac{\lambda }{{\rm{NA}}}$$

where *r* is the distance from the central maximum intensity pattern to the first dark pattern on the airy disk and *λ* is the wavelength of the light. That is, as the NA increases, the diffraction phenomenon decreases and the distance *r* also decreases. This means that the full width half maximum (FWHM) at the center maximum intensity distribution of the airy disk is reduced. At this time, this FWHM is called a circle of confusion (COC), which represents the optical spot where the rays passing through the lens can not be perfectly focused. In other words, as the NA of the lens increases, the COC decreases, which means that the resolving power of the lens to the image is higher. Since the maximum dioptric power of the B-EWLL was higher than that of the P-EWLL, the resolving power of the B-EWLL was superior, given equation (9). In addition, the B-EWLL had a wider viewing angle than that of the P-EWLL. In conclusion, the B-EWLL showed a small crosstalk ratio because the resolving power of the lenticular lens was high and the viewing angle was wide as compared with the P-EWLL.

## Results of 2D/3D switching operation and motion parallax observation test

**Figure 7:**
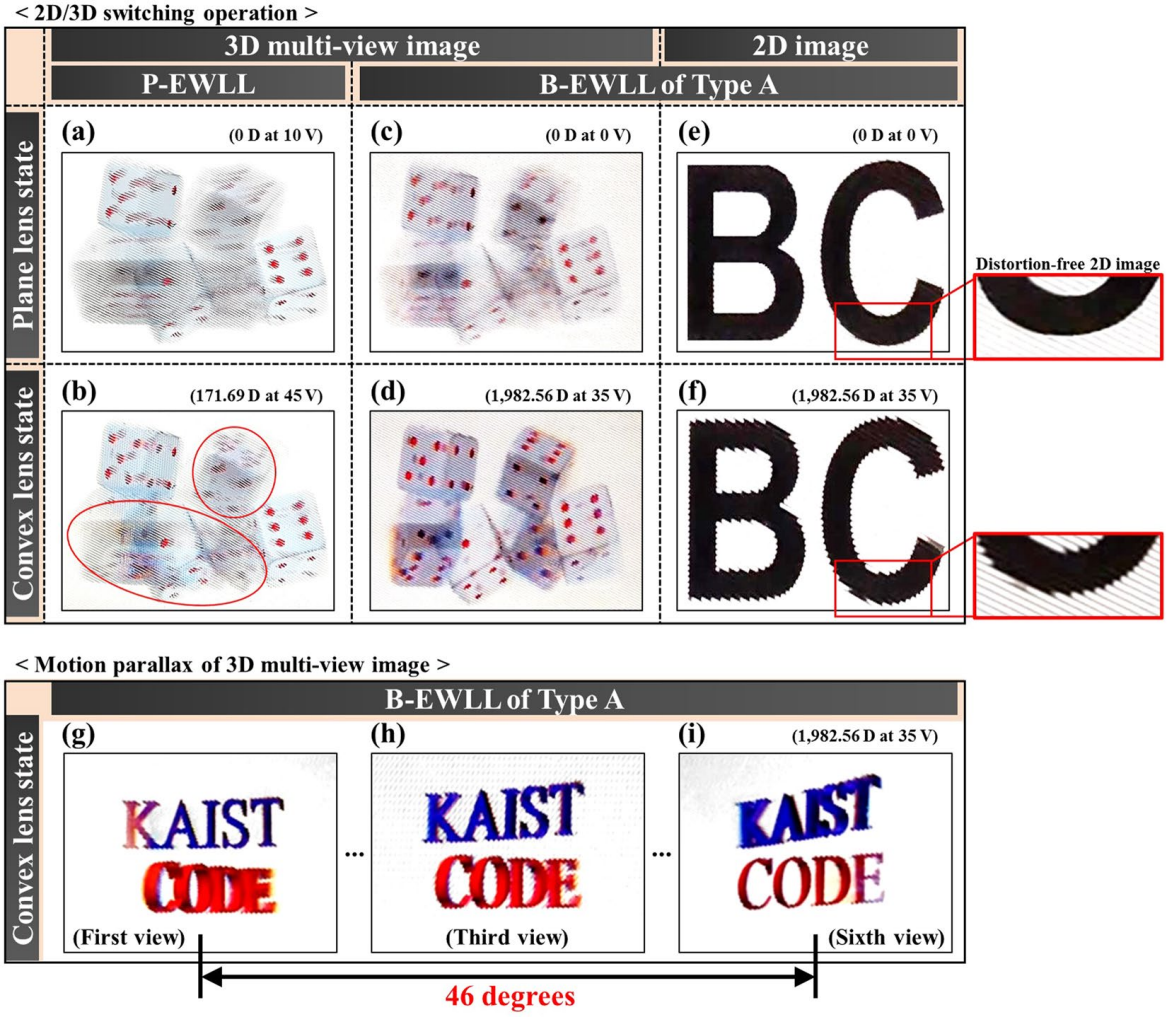
2D/3D switching operation test of the (**a**,**b**) P-EWLL and (**c**–**f**) B-EWLL of Type A. Motion parallax of 3D multi-view images from the (**g**–**i**) B-EWLL of Type A.

In this section, the results of viewing various images (2D image & 3D multi-view image) through the produced P-EWLL and B-EWLL of Type A were confirmed. First, a six-view dice image was used for the 3D multi-view image test. Figure 7a,b shows the results of a 3D multi-view image at different dioptric powers through the P-EWLL. At a voltage of 10 V, the P-EWLL had a plane lens state of 0 D, where no separation of images at each view point was observed, as shown in Fig. 7b. When the voltage of 45 V, which showed the maximum dioptric power of 171.69 D, was then applied, the multi-view image was separated to some extent, as shown in Fig. 7b. However, in the case of the image with the high disparity (red circle), almost no separation occurred and it was still difficult to confirm the appearance of the clean dice image. This was a result of failure to properly separate the 3D multi-view image by the view point because the dioptric power was low and the crosstalk ratio was also high.

Figure 7c,d present the results of checking the 3D multi-view image in the same way through the B-EWLL. Since the Type A B-EWLL had a dioptric power of 0 D at a voltage of 0 V, a 3D multi-view image in which images at each view-point were not separated was confirmed, as shown in Fig. 7c. After applying a voltage of 35 V, which showed a maximum dioptric power of 1,982.56 D, a clear separate image of the dice at a certain view point was identified, as shown in Fig. 7d. Since the images at high disparity were also completely separated, the shape of the dice could be recognized smoothly.

Next, Fig. 7e,f show the Alphabet 2D image through the B-EWLL. It was confirmed that the B-EWLL at 0 V has plane lens characteristics, as shown in Fig. 7d. On the other hand, it was confirmed that the 2D image is severely distorted by the convex lens state of the B-EWLL, as shown in Fig. 7f.

Finally, Fig. 7g,i show the results of observing the 3D multi-view image according to each view-point based on the viewing angle. For the measurement, the B-EWLL was placed on the six-view words image listing the words “KAIST” and “CODE”. By making a six-view image of words rather than pictures, it is easy to distinguish images at each view point. According to the above measurement results, the B-EWLL had a viewing angle of 46 degrees at 1,982.56 D, and the image was observed by rotating the camera based on that viewing angle information. When the six-view images were observed in the main field of view corresponding to the viewing angle of 46 degrees, a natural motion parallax was perceived. This was due to the high dioptric power and low crosstalk ratio of the B-EWLL, which allowed images to be clearly separated at each viewpoint. In conclusion, it was confirmed that the B-EWLL can show a natural 3D multi-view image.

## Conclusions

In this research, the B-EWLL was newly introduced. The intent of this study was to further improve the 3D performance of the conventional P-EWLL, which is lower than that of a commercial solid lenticular lens. For convenience of the fabrication process, a method of dosing ETPTA into a PMMA lenticular lens chamber was adopted. When UV curing of ETPTA was completed, the bottom of the chamber formed a spherical surface. It was confirmed that dosing 0.010 g of ETPTA was the most optimal condition for the B-EWLL. The B-EWLL showed considerable improvement over the various characteristics compared to the P-EWLL. First, the dioptric power was greatly improved from 171.69 D to 1,982.56 D. In addition, the viewing angle was greatly expanded, from 26 degrees to 46 degrees, and the crosstalk ratio also decreased significantly, from 27.27% to 16.18%. These optical properties of B-EWLL are comparable to those of a commercial solid lenticular lens, which has fixed focal length. Thanks to these improvements, the B-EWLL showed fine 3D multi-view image quality. Specifically, since the images corresponding to each of the viewpoints were almost completely separated, a clean and fine 3D multi-view image could be observed. In addition, a natural motion parallax could be perceived in the main field of view corresponding to the viewing angle of 46 degrees. At 0 V, the B-EWLL had a dioptric power of 0 D. Therefore, it was possible to show a clean 2D image without any external voltage, and a distortion-free 2D image was observed. Finally, compared to the P-EWLL, the maximum dioptric power implementation voltage was reduced by 10 V for the B-EWLL. As a result, this novel B-EWLL technology is advantageous for mobile device application because it can reduce the power consumption significantly by reduction of the operation voltage. In conclusion, a novel biconvex structure electrowetting lenticular lens showed clear 2D/3D switchable operation and excellent 3D performance compared to a solid lenticular lens.

## Electronic supplementary material

Supplementary Information

## References

[1] Fattal D (2013). A multi-directional backlight for a wide-angle, glasses-free three-dimensional display. Nature.

[2] Dodgson NA (2005). Autostereoscopic 3D displays. Computer.

[3] Choi K, Kim H, Lee B (2004). Full-color autostereoscopic 3D display system using color-dispersion-compensated synthetic phase holograms. Optics Express.

[4] Smalley DE, Smithwick QYJ, Bove VM, Barabas J, Jolly S (2013). Anisotropic leaky-mode modulator for holographic video displays. Nature.

[5] Na JH, Park SC, Kim SU, Choi Y, Lee SD (2012). Physical mechanism for flat-to-lenticular lens conversion in homogeneous liquid crystal cell with periodically undulated electrode. Optics Express.

[6] Huang YP, Liao LY, Chen CW (2010). 2-D/3-D switchable autostereoscopic display with multi-electrically driven liquid-crystal (MeD-LC) lenses. Journal of the Society for Information Display.

[7] Limura Y (2015). Liquid-filled tunable lenticular lens. Journal of Micromechanics and Microengineering.

[8] Limura, Y. *et al*. Microfluidically tunable lenticular lens. Solid-State Sensors, *Actuators and Microsystems (Transducers & Eurosensors XXVII), 2013 Transducers & Eurosensors XXVII: The 17th International Conference on. IEEE*, 1787–1790, 10.1109/Transducers.2013.6627135 (2013).

[9] Kim C (2016). Electrowetting lenticular lens for a multi-view autostereoscopic 3D display. IEEE Photonics Technology Letters.

[10] Lee J (2016). Improving the performance of an electrowetting lenticular lens array by using a thin polycarbonate chamber. Optics Express.

[11] Lippmann G (1875). Relation entre les phenomenes electriques et capillaires. Ann. Chim Phys..

[12] Berge B (1993). Electrocapillarite et mouillage de films isolants par l’eau. Comptes rendus de l’Académie des sciences. Série 2, Mécanique, Physique, Chimie, Sciences de l’univers, Sciences de la Terre.

[13] Berge B, Peseux J (2000). Variable focal lens controlled by an external voltage: An application of electrowetting. The European Physical Journal E.

[14] Kim J (2017). A fabrication method of opened structures for uniform liquid dosing in liquid lenticular systems. MOEMS and Miniaturized Systems XVI. International Society for Optics and Photonics.

[15] Harris WF (1997). Dioptric power. Its nature and its representation in three-and four-dimensional space. Optometry and vision science: official publication of the American Academy of Optometry.

