# Supplementary material for: Novel biconvex structure electrowetting liquid lenticular lens for 2D/3D convertible display
Source: Sci Rep. 2018 Oct 18;8:15416. doi: 10.1038/s41598-018-33983-x (PMC6194056; doi:10.1038/s41598-018-33983-x)
Supplement: Supplementary file 1 — Supplementary Information [file 41598_2018_33983_MOESM1_ESM.docx]

**Supplementary Information for ‘Novel biconvex structure electrowetting liquid lenticular lens for 2D/3D convertible display’**

# Jee Hoon Sim1,+, Junoh Kim1,+, Cheoljoong Kim1, Dooseub Shin1, Junsik Lee1, Gyohyun Koo1, Gyu Suk Jung1, and Yong Hyub Won1,*


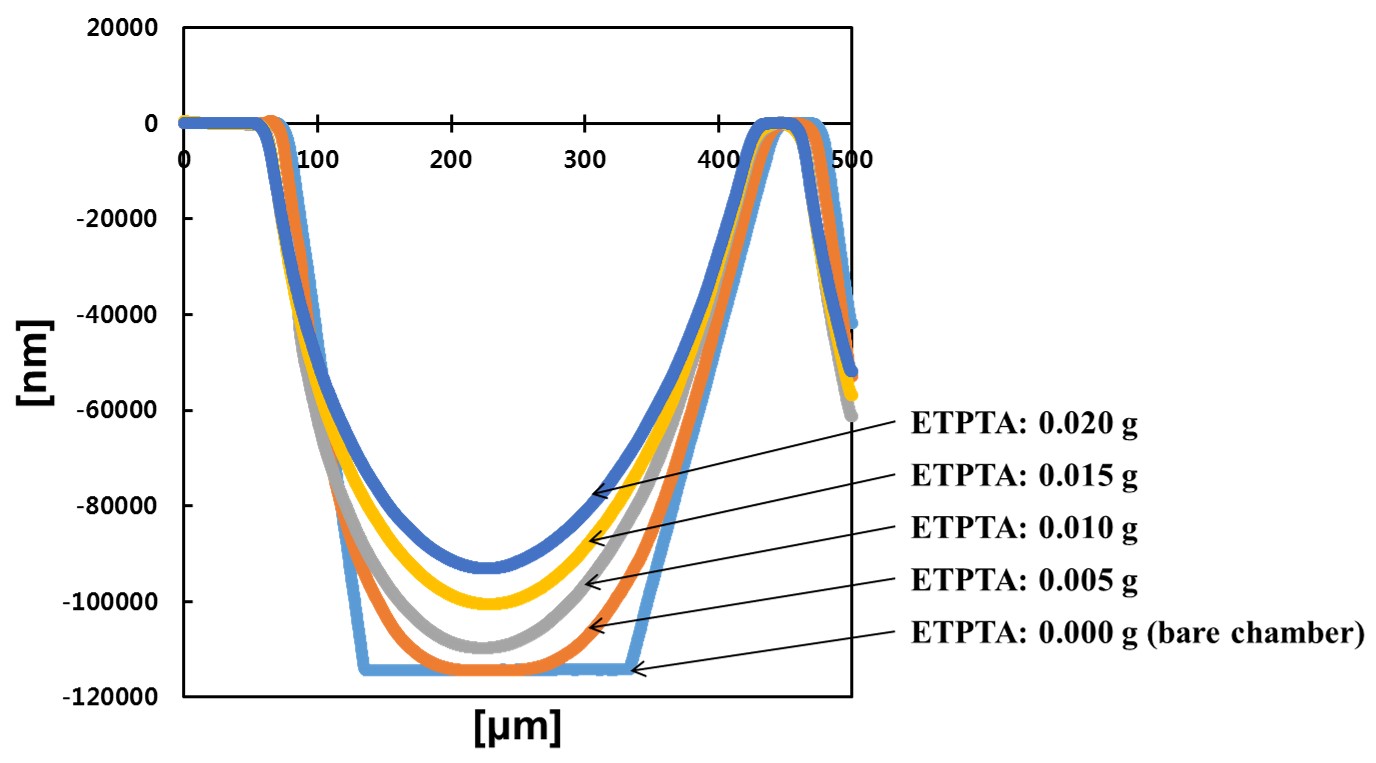


Figure S1. α-step measurements of PMMA lenticular lens chambers dosing ETPTA one time (0.005 g), two times (0.010 g), three times (0.015 g), and four times (0.020 g), respectively.


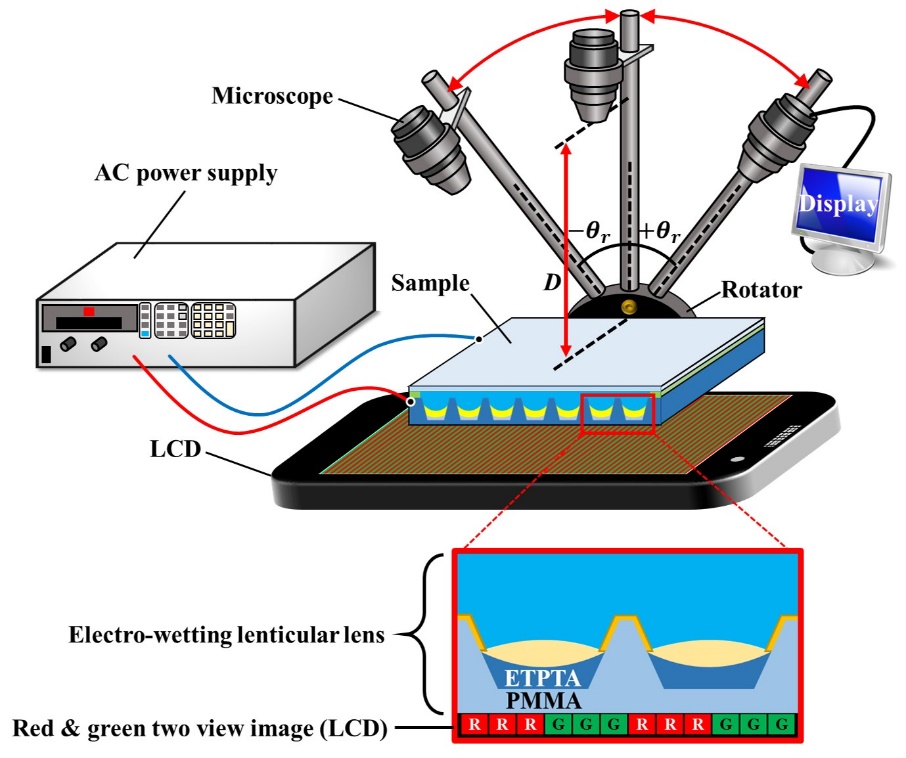


Figure S2. A schematic diagram of the viewing angle and crosstalk measurement of an electrowetting lenticular lens.
